# Pericarpial nectary-visiting ants do not provide fruit protection against pre-dispersal seed predators regardless of ant species composition and resource availability

**DOI:** 10.1371/journal.pone.0188445

**Published:** 2017-12-06

**Authors:** Priscila Andre Sanz-Veiga, Leonardo Ré Jorge, Santiago Benitez-Vieyra, Felipe W. Amorim

**Affiliations:** 1 Laboratório de Ecologia da Polinização e Interações–LEPI, Programa de Pós-graduação em Ciências Biológicas (Botânica), Instituto de Biociências, Universidade Estadual Paulista “Júlio de Mesquita Filho”, Botucatu, São Paulo, Brazil; 2 Departamento de Biologia animal, Universidade Estadual de Campinas, Campinas, São Paulo, Brazil; 3 Instituto Multidisciplinario de Biología Vegetal (CONICET), Universidad Nacional de Córdoba, Ciudad de Córdoba, Córdoba, Argentina; 4 Laboratório de Ecologia da Polinização e Interações–LEPI, Departamento de Botânica, Instituto de Biociências, Universidade Estadual Paulista “Júlio de Mesquita Filho”, Botucatu, São Paulo, Brazil; Indian Institute of Science, INDIA

## Abstract

Extrafloral nectaries can occur in both vegetative and reproductive plant structures. In many Rubiaceae species in the Brazilian Cerrado, after corolla abscission, the floral nectary continues to secret nectar throughout fruit development originating post-floral pericarpial nectaries which commonly attract many ant species. The occurrence of such nectar secreting structures might be strategic for fruit protection against seed predators, as plants are expected to invest higher on more valuable and vulnerable parts. Here, we performed ant exclusion experiments to investigate whether the interaction with ants mediated by the pericarpial nectaries of *Tocoyena formosa* affects plant reproductive success by reducing the number of pre-dispersal seed predators. We also assessed whether ant protection was dependent on ant species composition and resource availability. Although most of the plants were visited by large and aggressive ant species, such as *Ectatomma tuberculatum* and species of the genus *Camponotus*, ants did not protect fruits against seed predators. Furthermore, the result of the interaction was neither related to ant species composition nor to the availability of resources. We suggest that these results may be related to the nature and behavior of the most important seed predators, like *Hemicolpus abdominalis* weevil which the exoskeleton toughness prevent it from being predated by most ant species. On the other hand, not explored factors, such as reward quality, local ant abundance, ant colony characteristics and/or the presence of alternative energetic sources could also account for variations in ant frequency, composition, and finally ant protective effects, highlighting the conditionality of facultative plant-ant mutualisms.

## Introduction

Ant-plant defensive mutualisms represent an indirect defense strategy widely distributed among angiosperms in which plants provide energetic resources and/or housing for ants that in turn provide protection against herbivores [1–4]. Even though ant-plant associations range from facultative to obligate (reviewed in [5, 6]), most ant-plant associations are facultative [3, 5] and associations with defensive ants mediated by extrafloral nectaries (EFNs) represent a classic example of such non-obligatory interaction [1, 7, 8].

Extrafloral nectar is produced in secretory glands, generally not involved in pollination [1, 9]. Extrafloral nectar-visiting ants can protect plants by deterring or preying upon leaf [7, 10], bud and flower herbivores [8, 11–13], as well as pre-dispersal seed predators [14, 15]. However, some studies have suggested that EFN-visiting ants may be ineffective in plant defense [16–21] or even have negative effects by repelling pollinators or the natural enemies of herbivores [16, 22–26]. Hence, the outcome of plant-ant interactions may vary along a continuum from positive to negative effects [6, 27]. Thus, the net result of these interactions may depend on many factors such as ant density and herbivore abundance [28, 29]; ant behavior [12, 18, 21, 30–32]; herbivore vulnerability to ant predation [17, 20, 25, 28, 33, 34]; plant traits and resource abundance [25, 29, 35–39], as well as the local abiotic conditions [32, 40–43].

Although EFNs are present in both vegetative and reproductive structures [6], according to the predictions of optimal defense theory [44], plants are expected to invest more in protection of the most vulnerable and valuable parts, such as young leaves, buds and flowers [45, 46]. The occurrence of EFNs on reproductive structures may represent one example of such investment, as these parts are directly related to plant reproduction [47]. EFNs located on reproductive parts, such as the pedicel, sepals and bracts can extend their activity throughout the whole plant reproductive stage and attract ants to protect buds, flowers and developing seeds [14, 16, 45, 47, 48]. Moreover, plants can also present EFNs on fruits, and such nectaries can be derived from persistent floral nectaries [15, 49–51], or originated from newly developed structures [45, 52, 53]. In the first case, the so called post-floral pericarpial nectaries (PPNs) may be related to fruit protection against pre-dispersal seed predators. However, the few studies that have so far investigated the effectiveness of the association between PPNs and ants on fruit protection have provided contrasting results [15, 50, 54].

**Fig 1:**
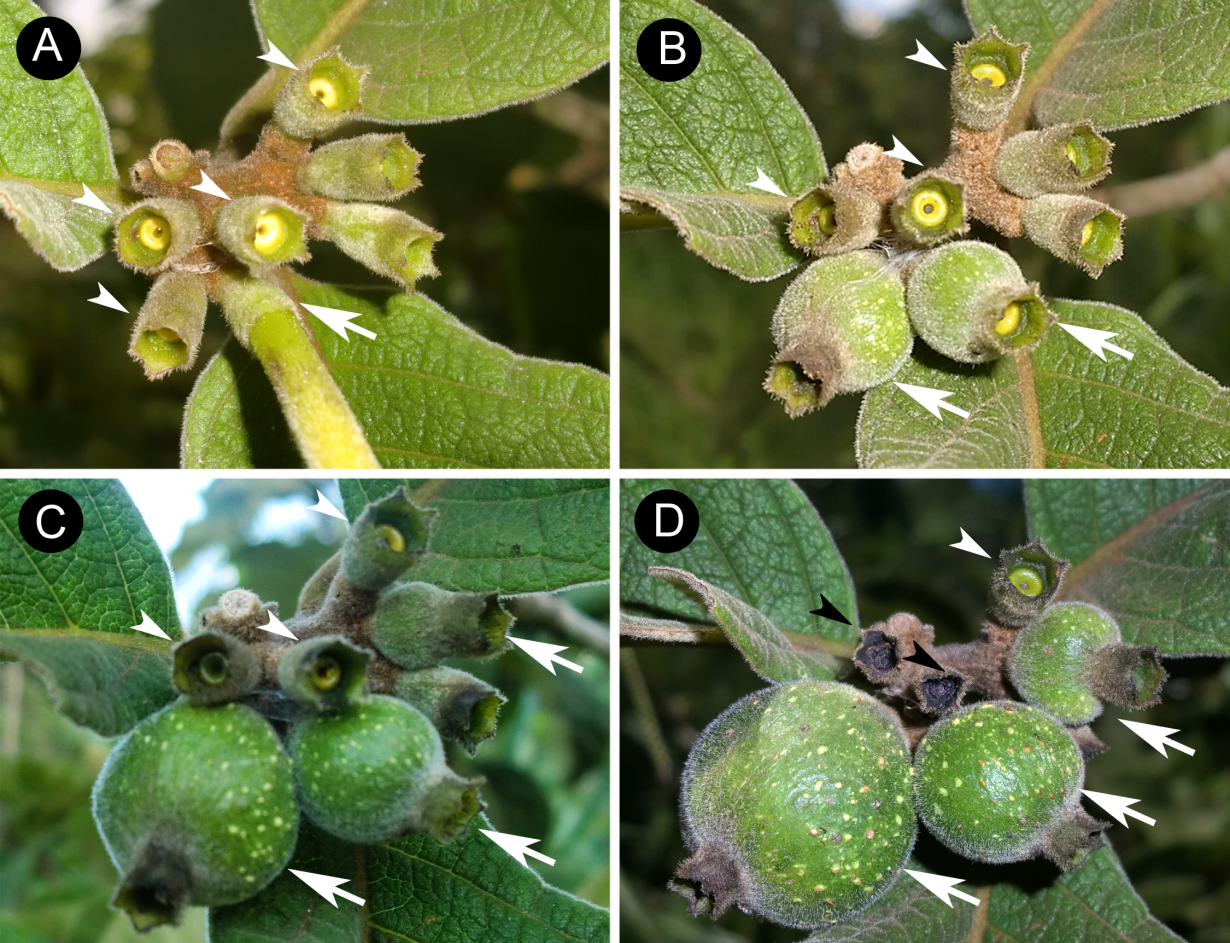
Post-floral pericarpial nectaries of *Tocoyena formosa* at different developmental stages. (A) Active post-floral nectaries just after corolla abscission (arrow heads). Note the corolla tube of a flower before abscission (arrow). (B) Post-floral pericarpial nectaries (PPNs) at the initial stage of development after one month of flower pollination (arrows) and post-floral nectaries derived from non-pollinated flowers (arrow heads). (C) PPNs on developing fruits two months after flower pollination (arrows), and post-floral nectaries derived from non-pollinated flowers (arrow heads). (D) Fruits at advanced stages of development (arrows) and a still active post-floral nectary after three months of corolla abscission of a non-pollinated flower (white arrow head), and senescent post-floral nectaries at the same age (black arrow heads).

In many plant species of the family Rubiaceae, floral nectaries continue secreting nectar after flower senescence and corolla abscission, originating PPNs [e.g., 49–51]. However, although many studies have demonstrated that ants can effectively protect EFN bearing plants against herbivores [8, 10, 12, 13], the ecological significance of PPNs on plant reproduction is poorly known [50, 54]. *Tocoyena formosa* (Cham. & Schlechtd.) K. Schum. (Rubiaceae) is a common and widely distributed shrub in the Brazilian Cerrado [55]. The species produces fruit-bearing PPNs which are constantly visited by many ant species, especially aggressive ants such as those from the genera *Ectatomma* and *Camponotus* [54]. In contrast to the small foliar EFNs commonly found in many Cerrado plants, PPNs of *T*. *formosa* produce copious amounts of nectar throughout fruit development. Moreover, even ovaries from non-pollinated flowers continue to produce nectar for about 2–3 months after corolla abscission (Fig 1).

In this study we tested whether PPN-visiting ants have positive effects on *T*. *formosa* reproductive success and whether the outcome of plant-ant interaction depends on ant species composition and resource availability. We hypothesized that *T*. *formosa*’s PPN-mediated interaction with aggressive ant species may provide fruit protection against pre-dispersal seed predators. However, since reward availability may influence the outcome of plant-ant interaction [56, 57] and the number of nectaries is highly variable among *T*. *formosa* plants, both ant species composition and visitation frequency may also be affected by nectar availability. In this sense, we also hypothesize that plants with more nectaries will receive more visits from competitively superior aggressive ant species with higher defensive abilities [30, 58], which may result in higher reproductive success in terms of fruit and seed set.

## Material and methods

### Study site and species characterization

The study was carried out in a private reserve of cerrado vegetation at the Palmeira da Serra farm (22°48’50” S, 48°44’40” W), located in the municipality of Pratânia, São Paulo state, southeastern Brazil. The reserve is about 720 m a.s.l. and comprises a total area of 224 ha. The climate is warm temperate (Cwa according to Köppen–Geiger) and markedly seasonal, with a dry winter from March to September and a hot summer from October to April. The mean annual temperature is 21°C and annual precipitation is around 1450 mm [59].

*Tocoyena formosa* is a deciduous shrub (< 3 m), whose leaf shedding occurs during the driest months (June to August) and new leaves sprout throughout the spring (September-October). Flowers are produced in inflorescences at the apex of branches and flowering occurs in November-January, and fruiting may extend until May. Flowers are tubular and the length of the corolla tube ranges between 60 and 150 mm. Pollination and sexual reproduction of *T*. *formosa* relies exclusively on long-tongued hawkmoths [60, 61].

### Ant effectivity on fruit and seed protection

In order to test whether ants effectively defend fruits against herbivores and seed predators, we conducted exclusion experiments that allowed us to test the effect of ants on plant reproductive success (i.e., fruit and seed set) within plants. In December 2014, we tagged 42 randomly marked plants. In each plant we tagged two branches with flower buds and randomly assigned them either as ant exclusion (treatment) or free ant access (control). Ants were prevented from accessing the PPNs by applying a nontoxic resin (Tanglefoot^TM^) on the branch base. Branches or leaves that could be used as bridges by ants to access the experimental branches were removed. We assessed ant efficacy by comparing the fruit and seed set between ant excluded and free ant access branches. We compared the number of fruits at the initial stage of development (hereafter initiated fruits), the number of fully developed fruits at the pre-mature stage (hereafter developed fruits) and the mean number of fully developed seeds per fruit. At the end of the fruiting season (from April to May of 2015) developed fruits were collected and kept in plastic containers for approximately one month until the emergence of the pre-dispersal seed predators. In addition, we checked all the fruits for the presence of dead larvae and adults that did not emerge.

To test the influence of the number of nectaries on ant efficacy, we quantified the total number of nectaries of each individual plant. We considered the total number of flowers as a proxy for nectary abundance, since those nectaries from ovaries that did not develop into fruits remained active during the development period of the adjacent fruits (see Fig 1).

### Plant reproductive success

We evaluated plant reproductive success using the rate of initiated fruits (number of initiated fruits / total number of flowers per branch), rate of developed fruits (number of developed fruits / number of initiated fruits per branch) and seed set (mean number of seeds produced per fruit). The mean number of seed per fruit was obtained from 2 to 5 developed fruits in those fruits from which seed predators were reared. Only fully developed and intact seeds were counted.

### Ant fauna and natural history observations

To assess the PPN-visiting ant fauna, we performed weekly censuses of ant activity on control branches of all tagged plants from February to April of 2015. The observations were carried out between 08h00 and 18h00 and lasted 2 minutes per plant. On average, we performed 10 censuses per plant, totaling 14 hours of observations. Ants were collected, preserved in 70% ethanol, and identified with the aid of specialists. Voucher specimens were deposited in the insect collection of the Pollination Ecology and Interactions Laboratory (LEPI) at the São Paulo State University, Unesp, Botucatu–SP.

We determined the frequency of visits of each ant species to the tagged branches by dividing the number of times a certain species was recorded in the branch by the total number of censuses made. Then, we considered as dominant those ant species that had the highest visitation frequency value in a given individual plant. While recording ant visitation, we also characterized their behavior by describing the activity of each species around the nectaries, as well as their response during the encounters with pre-dispersal seed predators.

### Pre-dispersal seed predator fauna

Reared insects were preserved in 70% ethanol and classified at the lowest taxonomic level possible. Voucher specimens were deposited in the insect collection of the LEPI. *Hemicolpus abdominalis* Hustache 1938 (Coleoptera: Curculionidae) voucher specimens were also deposited in the zoology collection of the São Paulo University (USP), São Paulo–SP, Brazil.

We assessed the relative frequency of each group of pre-dispersal seed predators on control branches (free ant access). Fruit infestation by insects was estimated through the total number of seed predators reared divided by the number of fruits collected. We estimated infestation for the overall seed predators and for each taxonomic group separately. We also observed the behavior of the pre-dispersal seed predators in the field, in order to describe their foraging and feeding habits in natural conditions, as well as their response to encounters with ants.

### Analyses

To test the effect of ants on seed predators and plant reproductive success (i.e., proportion of initiated fruits, developed fruits and the mean number of seeds per fruit), we used generalized linear mixed models (GLMM). For this, we used a Gaussian model for the quantitative response variables (i.e., number of seeds per fruit, number of seed predators per fruit and number of weevils per fruit) and binominal models for proportions of fruits started and developed. In all cases, treatments (ant absence and presence) were considered as fixed effects and plant individuals were assigned as the random effect. For each response variable we used the Akaike Information Criterion (AIC) to compare a model including both fixed (ant absence and presence) and random (individual plant) effects with a null model with only the random effect (individual plant). For model selection, we considered the lowest AIC values to best fit the data [62]. To test whether the effect of ants on the number of seed predators per fruit depend on dominant ant species, we used a GLMM in which plant individuals were assigned as random factors, while treatments (ant absence and presence) and the dominant ant species were used as fixed effects. We used the mean number of seed predators per fruit as the response variable. We also used the model selection approach to test whether including the dominant ant species in each plant improved model prediction. Through AIC we compared a model including all fixed effects (ant exclusion treatment and dominant ant species) as well as the interaction between them, a model including all fixed effects, but with no interaction between them, a model including only the treatment (ant absence and presence) as fixed effect, and a null model with only the intercept as fixed effect. For all models plant individuals were included as a random factor.

To test the hypothesis that plants with higher number of nectaries are more visited by ants, we used a simple linear regression model with the total number of nectaries (i.e., total number of flowers produced per plant) as predictor variable and the frequency of the PPN-visiting ants as dependent variable. To test whether resource availability influenced the effect of ants on plant reproductive success, we used both GLMM and model selection approaches. We fitted Gaussian models to predict the mean number of seeds per fruit. In all models plant individuals were considered as random effect and the number of nectaries and ant treatments (ant absence and presence) as fixed effects. By means of AIC we compared among a model with all fixed effects, as well as the interaction between them, a model including all fixed effects but with no interaction between them, a model with only the ant treatment as the fixed effect, a model with only the number of nectaries as fixed effect, and a null model containing only the random effect (individual plant).

In order to assess sample completeness of the PPN-visiting ant fauna, we calculated sample-based rarefaction and extrapolation curves based on Hill numbers of the diversity order of *q* = 0 (species richness; see [63]). To perform this analysis, we used each 2 minutes’ observation session as a sample unit, and then, we extrapolated our reference sample up to the double of the number of observation sessions. Following Hsieh et al. [64] we used a bootstrap method with 250 replications to obtain 95% confidence intervals for both the rarefied and extrapolated curves. The iNEXT package in R programming language was used to perform rarefaction and extrapolation analyses [64]. Also, to explore the occurrence of different ant composition among plants (N = 42 plants), we performed a Non-metric Multidimensional Scaling (NMDS) based on the frequency of PPN-visiting ants in each plant using Bray-Curtis distances. To compute the NMDS we used the function metaMDS in vegan package of R software [65]. In this analysis we separated plants in three groups based on the dominant ant species, *i*.*e*., plants dominated by *Ectatomma tuberculatum*, plants dominated by species of the genus *Camponotus* (regarded as a single functional group) and plants dominated by other ant species. Since the number of nectaries per plant may affect the composition of the ant fauna, hence the occurrence of a given dominant ant, we used nectary as an environmental variable to fit surfaces of nectary abundance to the resulting NMDS ordination. To fit smooth surfaces over the NMDS ordination, which allows better visualizing the effect of the number of nectaries on the ant fauna composition, we used the function ordisurf in vegan package in R, which applies generalized additive models in gam function (see details in [65]). Finally, in order to test whether the number of nectaries affects the ant composition among plants, we performed a permutational multivariate analysis of variance (PERMANOVA), which test for significant differences in the average distances between plants. To perform the PERMANOVA test we used the adonis*2* function in vegan package in R [65].

To assess the effect of pre-dispersal seed predators on seed set, we performed a simple linear regression analysis to test for the relationship between the total number of seed predators per fruit (including all groups of seed predators) and the mean number of seeds per fruit. But, since the weevil *H*. *abdominalis* and wasps revealed to be the main pre-dispersal seed predators of *T*. *formosa*, we also used multiple linear regression analysis to test for the relationship between the number of weevils and wasps as independent predictors of the number of seeds per fruit

## Results

### Ant effect on seed predators and plant reproductive success

Ants did not affect fruit production or seed set. In all cases the null model, which considered only the random effect of individual plant, presented the lowest AIC value (Table 1). Thus, neither the proportion of fruits started (ant absence: 0.46 ± 0.20; presence: 0.47 ± 0.16) nor the proportion of fruits developed (ant absence: 0.89 ± 0.17; presence: 0.91 ± 0.13) were affected by ants. Moreover, we found no differences in the mean number of seeds produced per fruit in branches with (39.83 ± 14.16) and without ants (39.44 ± 17.69). Fruits from branches with or without ants also presented similar levels of infestation by pre-dispersal seed predators (branches with ants: 1.30 ± 1.62; branches without ants: 1.25 ± 1.32). Even the occurrence of weevils was not affected by ants (ant absence: 0.64 ± 0.58; ant presence: 0.59 ± 0.62). Finally, the dominant ant species also had no effect on the number of seed predators per fruit in each plant. The null model (considering only the random effect of individual plant) always performed better than the ones that included both the effect of presence/absence of ants and ant identity (Table 2). Also, we found no influence of the total number of nectaries per plant on ant visitation frequency (R^2^ = 0.03, *p* = 0.123) and no ant effect on the seed set regardless of the number of nectaries per plant. Models not accounting for the ant effect on the plant reproductive success performed better than those that included ant treatment as a factor (Table 3). Overall, our results strongly suggest that ants had no effect on plant reproductive success.

**Table 1:** Models tested for ant effect on seed predators and plant reproductive success. Comparison of models including the effects of the ant treatment (ant absence and presence) and a null model with only the random effect (individual plant).

Models	AIC	ΔAIC	d.f.
**Seed predators per fruit**			
Model with treatment	303.5	2.2	4
Null Model	301.6	0.0	3
**Weevils per fruit**			
Model with treatment	157.3	2.0	4
Null Modell	155.5	0.0	3
**Proportion of initiated fruits**			
Model with treatment	397.4	1.8	3
Null Model	395.7	0.0	2
**Proportion of developed fruits**			
Model with treatment	204.1	1.3	3
Null Model	203.0	0.0	2
**Mean number of seeds per fruit**			
Model with treatment	3389.5	1.7	4
Null Model	3387.8	0.0	3

**Table 2:** Models testing the effect of dominant ant species on the mean number of seed predators per fruit. Comparison of models including the effects of treatment (ant absence and presence), dominant ant species and the interaction between treatment and dominant ant species with a null model with only the random effect (individual plant).

Models	AIC	ΔAIC	d.f.
Treatment + Dominant ant species + Interaction	310.7	8.8	8
Treatment + Dominant ant species	306.6	4.7	6
Treatment	304.0	2.2	4
Null Model	301.7	0.0	3

**Table 3:** Ant exclusion experiment and nectary effect on the mean number of seeds per fruit. Comparison of models including the effects of treatment (ant absence and presence), nectary abundance and interaction between treatment and nectary with a null model with only the random effect (individual plant).

Models	AIC	ΔAIC	d.f.
Treatment + Nectaries + Interaction	705.7	2.7	6
Treatment + Nectaries	705.6	2.2	5
Treatment	706.7	3.1	4
Nectaries	703.6	0.0	4
Null Model	704.7	0.9	3

### PPN-visiting ant fauna

We recorded 10 ant species distributed in seven genera and five subfamilies: Ectatomminae: *Ectatomma tuberculatum* Olivier 1792; Formicinae: *Brachymyrmex* sp., *Camponotus ager* Smith 1858, *Camponotus crassus* Mayr 1862, *Camponotus renggeri* Emery 1894 and *Camponotus rufipes* Fabricius 1775; Myrmicinae: *Cephalotes* sp. and *Crematogaster goeldii* Forel 1903; Ponerinae: *Neoponera villosa* Fabricius 1804; Pseudomyrmicinae: *Pseudomyrmex gracilis* Fabricius 1804 (S1 Table). Rarefaction analysis showed sampling completeness of the *T*. *formosa* PPN-visiting ants, since the rarefied curve reached the asymptote after 250 samples and the extrapolated one revealed that no more species are expected at *T*. *formosa* PPNs (S1 Fig).

*Ectatomma tuberculatum* and the species of the genus *Camponotus* in turn, were the most frequent ants in the population of *T*. *formosa* with respectively 225 and 223 individuals observed in all plants. *Ectatomma tuberculatum* was recorded in 60% of the plants and was the dominant ant species in 55%, while *C*. *crassus* occurred in 19% of the plants, being dominant in only 14% of the plants. *Camponotus rufipes* was dominant in two plants, while *C*. *renggeri* and *Cephalotes* sp. were the dominant species in only one plant each. Among the small-sized ant species (1-3mm), *C*. *goeldii* and *Brachymyrmex* sp. were the most abundant ant species at all plants they occurred, though they were very uncommon in the whole population. Both species occurred in seven plants, but the first was the dominant species in six of them, while the second in only one plant. Among large- (7–15 mm) and medium-sized (4–7 mm) ants in turn, *N*. *villosa* was observed as a dominant species in only two plants, though presenting a very low abundance in both plants.

**Fig 2:**
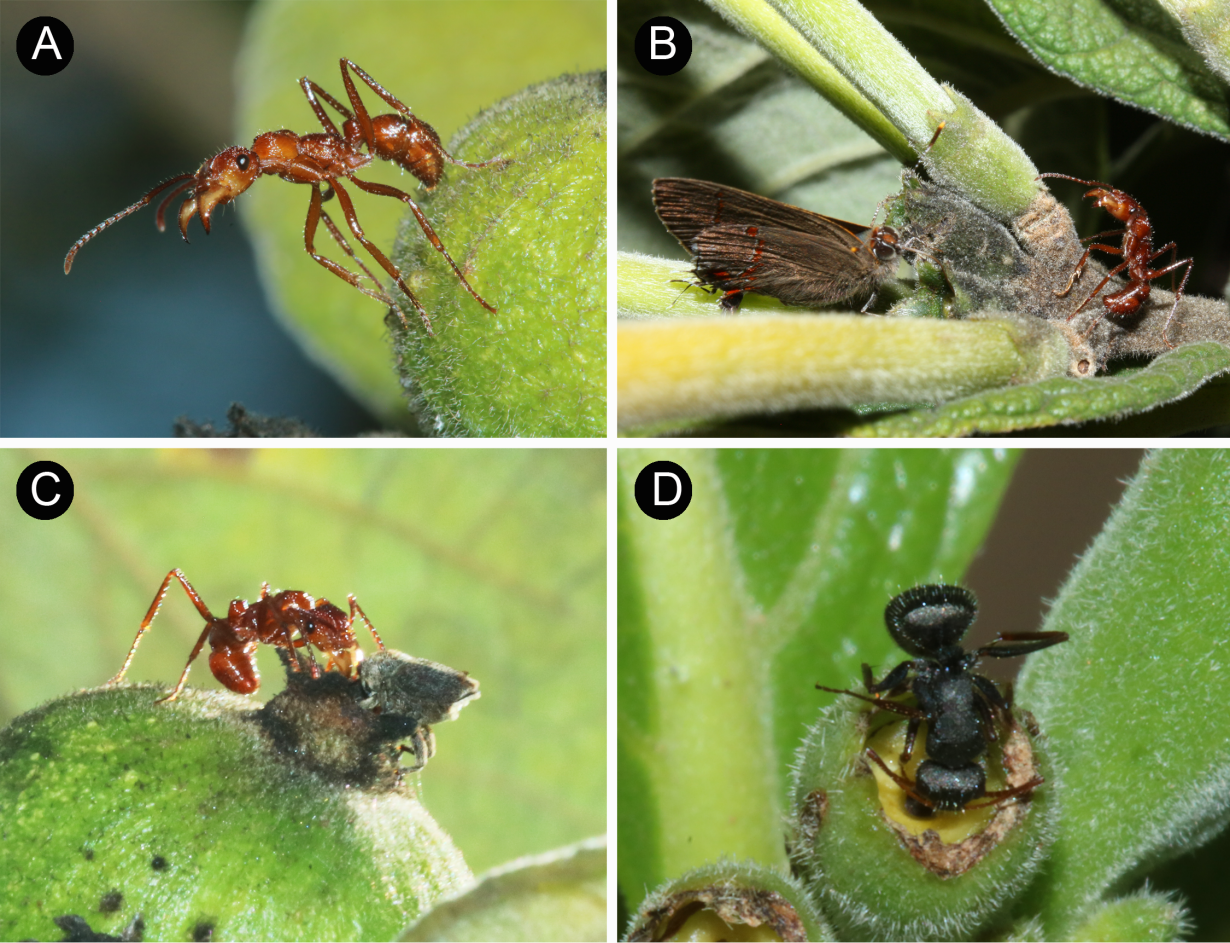
Some PPN-visiting ant species of *Tocoyena formosa*. **(**A) *Ectatomma tuberculatum* with its jaws open on a *T*. *formosa* fruit. (B) *E*. *tuberculatum* approaching a nectary where the butterfly *Calycopis* sp. (Lycaenidae) was visiting. The encounter between the ant and the butterfly did not result on agonistic interaction. (C) *E*. *tuberculatum* and *H*. *abdominalis* visiting the same nectary. (D) *Camponotus crassus* visiting a nectary.

*Ectatomma tuberculatum* was the most aggressive PPN-visiting ant, often seen standing on the fruits with their antennae and mandibles held wide open (Fig 2A). On some occasions this position preceded attacks to other insects which approached the fruits. We recorded antagonistic behavior toward flies and an effective predation of the main pre-dispersal seed predator *H*. *abdominalis*. However, in most cases this behavior did not result in prey capture. For instance, on some occasions *E*. *tuberculatum* totally ignored the presence of other insects (Fig 2B), including *H*. *abdominalis* weevil (Fig 2C). The second most abundant ant species *C*. *crassus* was more active when compared to *E*. *tuberculatum*, and also presented a more exploratory behavior on *T*. *formosa* branches and fruits (Fig 2D). Although *C*. *crassus* ants were not as aggressive as *E*. *tuberculatum*, they occasionally attacked flies on *T*. *formosa* fruits. The small-sized ants were recorded once attacking another ant species (*Cephalotes* sp.) and a Lepidoptera larva. However, this group of ants rarely displayed any agonistic interactions with other insects.

**Fig 3:**
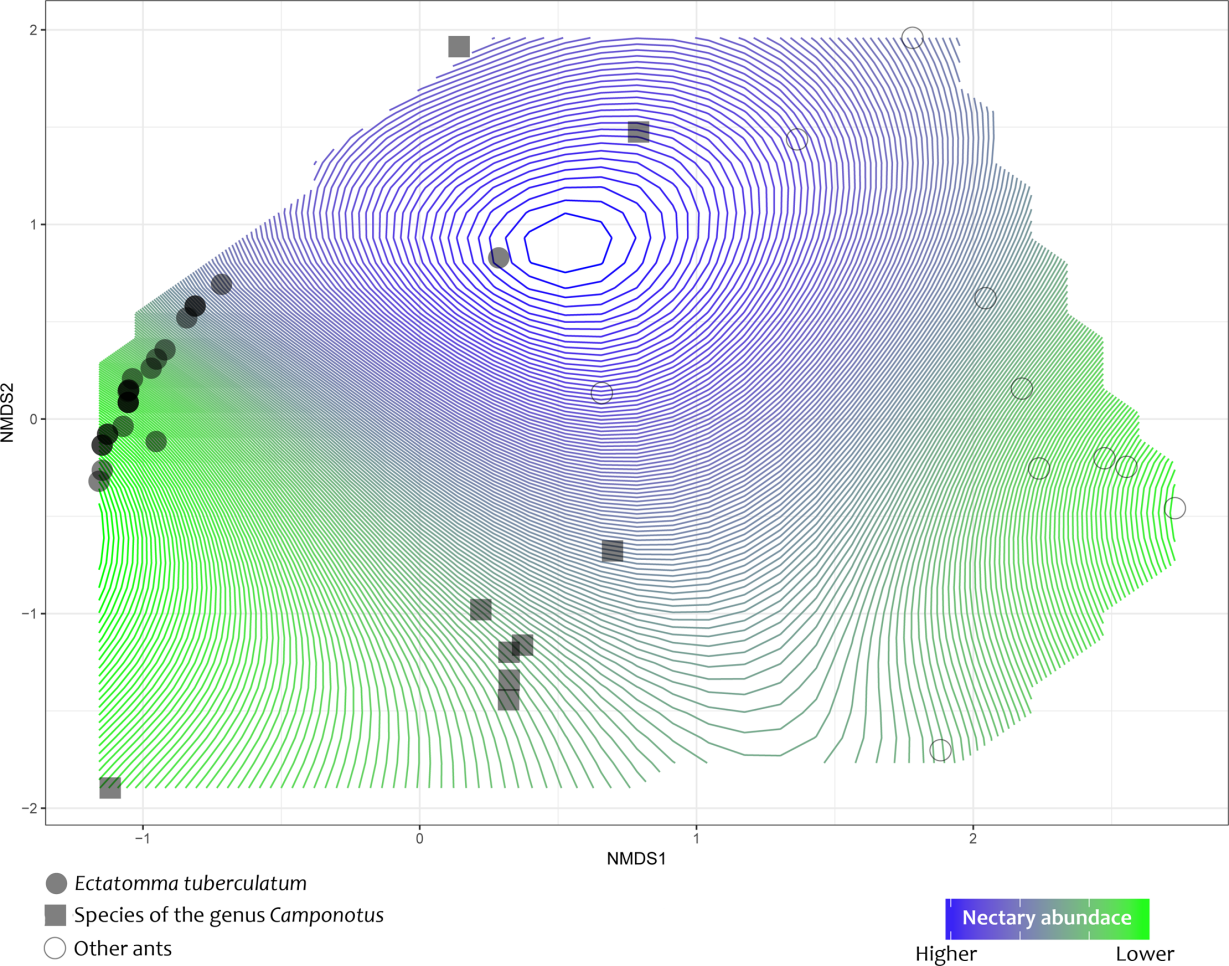
Nonmetric multidimensional scaling (NMDS) ordination of the ant fauna associated with *Tocoyena formosa* plants. Symbols on the NMDS ordination diagram represent the dominant ant species which occupy a given individual plant in the population. Surfaces of nectary abundance were fitted over the diagram (stress 0.0415, R^2^ = 0.99) to highlight the effect of the amount of resources (number of nectaries) on the composition of the ant fauna.

NMDS ordination showed that most differences in ant composition among plants were due to the occurrence of *E*. *tuberculatum* and species of the genus *Camponotus* as dominant ants (stress: 0.0415, R^2^ = 0.99; Fig 3). Plants in which these ants occurred were most similar in ant composition fauna. Generally, when one of these ant species was present in a given plant, few or no other ant species co-occurred. Furthermore, plants visited by the other recorded species did not show clear differences on ant composition (Fig 3). PERMANOVA revealed that the number of nectaries did not affect PPN-visiting ant composition fauna (Pseudo-*F*_1,40_ = 1.08, *p* = 0.339, 10,000 permutations).

### Pre-dispersal seed predator fauna

Hymenoptera (48.2%), Coleoptera (43.3%), Diptera (7.5%) and Lepidoptera (0.97%) were the main pre-dispersal seed predators found on the developing fruits of *T*. *formosa* (in 245 fruits from the control branches; S2 Table). Hymenoptera was represented mainly by the Eurytomidae micro wasp *Prodecatoma* sp., and the Curculionidae beetle *H*. *abdominalis*, which comprised 97% of all Coleoptera reared from fruits. Weevil beetles were observed in 69% of the plants, wasps in 48%, Diptera in 29% and Lepidoptera only in 7% of the plants. We found no relationship between the number of seed predators per fruit (considering all insect groups) and the mean number of seeds produced per fruit (R^2^ = 0.04, F_1,40_ = 2.79, *p* = 0.103). However, we found significant relationship between fruit infestation and seed set when considering only the two most important group of seed predators (weevil and wasp), which was explained mainly due to the negative correlation between the mean number of weevil beetles and the mean number of seeds per fruit (weevil: F_2,39_ = 6.76, *p* = 0.013; wasp: F_2,39_ = 0.69, *p* = 0.413).

**Fig 4:**
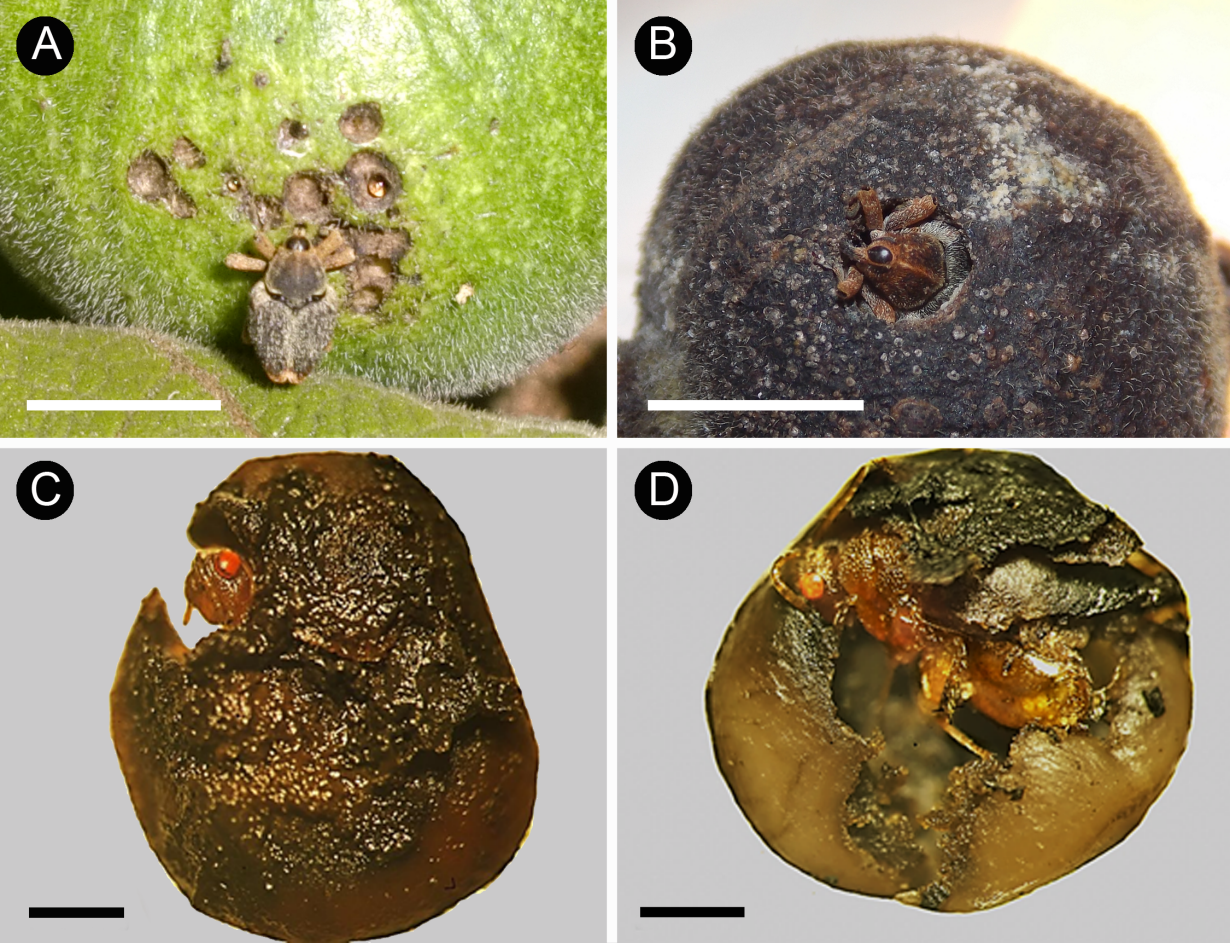
Main pre-dispersal seed predators of *Tocoyena formosa*. (A) The weevil *Hemicolpus abdominalis* perforating the fruit pericarp with its rostrum. (B) An adult of *H*. *abdominalis* emerging from a fruit by perforating the pericarp. (C and D) Adults of the wasp *Prodecatoma* sp. emerging from their hosting seeds. Scale of A and B: 5 mm; C and D: 1 mm.

*Hemicolpus abdominalis* is a diurnal weevil species which was frequently seen ovipositing on *T*. *formosa* fruits through small holes on the pericarp made with its mouth parts (Fig 4A). Weevil larvae fed on developing seeds and each larva usually consumed more than one seed per fruit. A single fruit could bear up to four weevil larvae, but in most cases fruits harbored only one or two. *Hemicolpus abdominalis* larvae pupated inside the fruit and after reaching the adult phase perforated the pericarp in order to leave the fruit (Fig 4B). Notwithstanding, fruits infested by weevil larvae usually reached full development. Wasp oviposition on fruits also occurred during daylight and their larvae fed exclusively on a single seed throughout their whole development (Fig 4C and 4D). We found up to 20 specimens of the wasp *Prodecatoma* sp. occurring in a single fruit. After they had developed into adults the wasps left the fruit by perforating its pericarp without causing fruit abortion. Moth larvae were the largest-sized pre-dispersal seed predators of *T*. *formosa*. These insects inflicted the greatest damage to *T*. *formosa* fruits, since infested fruits usually had 100% of the seeds consumed which eventually led to fruit abortion. Fruits infested by fly larvae also had most seeds and pulp consumed, often leading to fruit abortion.

## Discussion

Post-floral pericarpial nectary-attracted ants did not affect *T*. *formosa* reproductive success. Neither the large-sized and aggressive ants nor the abundance of nectaries affected ant performance in seed protection against pre-dispersal seed predators. Developing seeds suffered predation mainly due to the activity of weevils and wasp larvae. Although the infestation by these seed predators did not frequently result in fruit abortion, the larger the number of beetles per fruit, the lower the number of seeds produced, indicating that *H*. *abdominalis* is the most important pre-dispersal seed predator of *T*. *formosa*. To the best of our knowledge, this is the first study to report the behavior and feeding habits of this weevil [66], which might present host specific oviposition preferences.

Similarly to our results, Del-Claro et al. [50] found that the interaction with ants mediated by the PPNs of *Palicourea rigida*, another Rubiaceae species occurring at the cerrado vegetation, did not provide protection from pre-dispersal seed-consuming wasps, but ants had an indirect effect on plant reproductive success by protecting leaves against foliar herbivores. Though we did not test whether ants provide protection against foliar herbivory, in another population of *T*. *formosa* in the Minas Gerais state, southeastern Brazil, Santos and Del-Claro [54] did not find significant differences in foliar herbivory or in fruit production between plants with and without ants. However, in another system involving a completely different ant fauna visiting *Mentzelia nuda*, a Loasaceae species with a similar post-floral nectary to that of *T*. *formosa*, PPN-ant association was shown to be quite effective in seed defense against pre-dispersal predators, since there was a positive effect of ant presence on pod and seed formation [15].

Several other studies that also tested for the effect of ants associated with EFN located close to reproductive plant parts have reported contrasting results (see [6] and references therein). Inouye and Taylor [14] demonstrated that ants visiting EFN from involucral bracts of *Helianthella quinquenervis* (Asteraceae) reduced seed predation by tephritid and agromyzid flies. Similarly, in *Costus woodsonii* (Zingiberaceae) ants attracted to EFN from bracts on the inflorescence negatively interfered with fly oviposition on immature fruits [11]. In *Caryocar brasiliense* (Caryocaraceae), EFN-visiting ants have been shown to prevent phytophagous insects from ovipositing on both buds and fruits [13]. Nonetheless, O´Dowd and Catchpole [16] showed that ants attracted by EFN from capitula bracts of *Helichrysum* spp. (Asteraceae) did not reduce seed predation by insects. In *Chamaechrista nictitans* (Fabaceae), endophytic seed predators which larvae develop inside the fruit were shown to be unaffected by ant attacks [20]. Hence the efficacy of ants in protecting plants against herbivores may depend on the composition of the ant fauna [12, 18, 26, 67] and the herbivore fauna [17, 28], in conjunction with the nature of the plant-ant association [4, 28, 68, 69].

Extra floral nectary-associated ants and their benefits on plant reproductive success are reported to be temporally and spatially variable in the Brazilian Cerrado [29, 67, 70–74]. Despite this, the overall pattern of plant-ant mutualistic networks remains stable over time and space [71, 73, 75] due to the presence of competitively superior ant species in the core of such networks [58, 75]. In this ecosystem, species from the genera *Camponotus* and *Ectatomma* are among the most common and effective EFN-visiting ants [10, 12, 13, 32, 71, 75], and among them, *C*. *crassus* and *E*. *tuberculatum* are known as highly aggressive species [29, 76, 77]. Species of the geuns *Camponotus* are reported for their numerical dominance in most arboreal communities, which likely explains their position as core species within many plant-ant networks [71, 73, 78]. Additionally, some species from this genus present adaptations for feeding on liquid resources which allows the uptake of higher amounts of nectar [79]. *Ectatomma tuberculatum*, on the other hand, are less specialized in nectar consumption [73]. This ant, despite being very aggressive, is a primitive ant species which lacks a “social stomach” [76]. Such characteristic obligates *E*. *tuberculatum* to transport nectar between the jaws impairing it from attack herbivores and act as effective defensive mutualists (S2 Fig). Also, *E*. *tuberculatum* often stands at the peripheral position of many plant-ant networks in the Cerrado, highlighting the less specialized behavior of this ant on EFN-bearing plants [70, 73, 75]. Indeed, in another Cerrado area, *E*. *tuberculatum* was found to be more abundant in plants with exudate-producing insects rather than EFN-bearing plants [73]. Such characteristics of *Ectatomma* ants may explain the absence of plant protection against pre-dispersal seed predators, even in those *T*. *formosa* individuals in which *E*. *tuberculatum* occurred as the dominant ant species. It is noteworthy, however, that ant protection against pre-dispersal seed predators in *T*. *formosa* was ineffective even in those plants in which species of the genus *Camponotus* were the dominant ant species. In another population of *T*. *formosa* in which *C*. *crassus* was very abundant and also occurred in higher frequencies among plant individuals, this species also did not provide plant protection against both seed consumers and foliar herbivores [54].

Recently, Jones et al. [80] have shown that the presence of ants associated with EFN on pedicels of flowers and inflorescence did not interfere with pre-dispersal seed predation in *Senna mexicana* var. *chapmanii* (Fabaceae) and that only plants in sunny habitat were benefited by ant presence [43]. Additionally, EFN-visiting ants may negatively affect the plant fitness if they, for example, interfere with other predators of herbivores [20–22]. For instance, ants visiting the stipular EFN of *Vicia sativa* (Fabaceae) were shown to provide indirect protection to pod-boring herbivores (tortricid moths) against other predators and parasitoids [23]. Similarly, Lenoir and Pihlgren [81] found no defensive role of ants against seed predation by beetles (Coleoptera: Bruchidae) and that seed infestation tend to be lower when ants were excluded from plants of *Vicia sepium* (Fabaceae). Although one should not overlook the costs associated with EFN-attracted ants, we found no evidence that PPN-visiting ants provided protection to the pre-dispersal seed predators. Moreover, fruits from ant-excluded branches did not present lower infestation rate than fruits with free ant access.

Despite the fact that intrinsic differences in the amount of resources among plants can influence ant visitation frequency, and consequently the outcome of plant-ant interaction [35, 57, 74, 82], our results showed that ant visitation frequency was not affected by the number of nectaries. Variation in resource abundance among plants also had no effect on the composition of the ant fauna inhabiting *T*. *formosa* plants. Instead, few dominant ant species were the main visitors in most plants. This reveals that plants are dominated by competitively superior species [58]. Hence, at species level, the prevalence of large ants on most of *T*. *formosa* individuals supports the existence of links between plant traits and ant composition, since plant species with higher amounts of nectar may better fit the higher energetic requirements of large-sized and competitively superior ants [67]. Nonetheless, an alternative explanation that must be considered is that ant dominance can just reflect the local ant fauna composition, instead of interspecific competition for higher quality plant resources (see [73]).

In this context, it is worth considering that the identity and behavior of the most important seed predator may also have accounted for the absence of protection by ants, since specialized antagonists can present adaptations to escape indirect plant defenses [18,83,84], like *H*. *abdominalis* weevil which the exoskeleton toughness prevent it from being predated by most ant species. On many occasions, ants failed to notice insects located at the base of the fruit while they were visiting the PPNs at the top of it. However, it is worth mentioning that *E*. *tuberculatum* response to encounters with *H*. *abdominalis* varied from aggressive attacks to totally ignoring weevil´s presence, even when they were placed at the same nectary.

From the above considerations we suggest that lack of ant protection in our study system may be related to the overall conditionality of facultative mutualisms [69]. The low interaction intimacy of this facultative plant-ant association, i.e., ants do not rely exclusively on *T*. *formosa* for energetic provision, predicts that the interaction outcome can be highly variable [4, 42, 85]. Even though *T*. *formosa* apparently invests in high amounts of nectar (as the PPNs produce nectar for at least four moths throughout the whole fruit development), this plant-ant association does not appear to represent an exception from the notion that facultative plant-ant interactions are highly variable [69].

Our observations could also indicate that characteristics of *H*. *abdominalis*, the most important seed predator, may provide protection against ant attacks. On the other hand, at the community level, yet to be explored factors, such as reward quality and attractiveness [22, 38], local ant abundance [18, 86], ant colony characteristics [56, 76], abiotic factors [40–43] or the presence of alternative energetic sources [35, 56, 73] could also account for variations in ant frequency, composition and ant protective effects [46, 58, 72]. Furthermore, an interesting approach would be to investigate whether this lack of protection entails costs for plants, i.e., balance of costs and benefits of the plant-ant association (e.g., [82], reviewed in [27]) and whether these results are consistent in space and time.

## Supporting information

S1 Table Ant species observed visiting PPNs on control branches of *Tocoyena formosa*. Total ant abundance, Mean abundance (±SD) per census, mean visitation frequency (±SD).
(DOCX)

S2 Table Pre-dispersal seed predators of *Tocoyena formosa*. Abundance, relative frequency and mean number (±SD) of seed predators per fruit (n = 245 fruits) among *Tocoyena formosa* individual plants.
(DOCX)

S1 Fig Sample-based rarefaction and extrapolation curves with Hill numbers of the diversity order of q = 0 for the *Tocoyena formosa*’s PPN-visiting ant fauna. Solid and dotted lines represent rarefaction and extrapolation curves, respectively. Shaded area shows the 95% confidence intervals after 250 bootstraps.
(TIF)

S2 Fig *Ectatomma tuberculatum* visiting *Tocoyena formosa* pericarpial nectaries. (A) and (B) show *E*. *tuberculatum* carrying a nectar droplet between its jaws (arrows).
(TIF)
