# Supplementary material for: Pericarpial nectary-visiting ants do not provide fruit protection against pre-dispersal seed predators regardless of ant species composition and resource availability
Source: PLoS One. 2017 Dec 6;12(12):e0188445. doi: 10.1371/journal.pone.0188445 (PMC5718428; doi:10.1371/journal.pone.0188445)
Supplement: S1 Table — Total ant abundance, Mean abundance (±SD) per census, mean visitation frequency (±SD). (DOCX) [file pone.0188445.s001.docx]

**S1 Table. Ant species observed visiting PPNs on control branches of *Tocoyena formosa*.** Total ant abundance, Mean abundance (±SD) per census, mean visitation frequency (±SD).

| **Ant Species** | **Total Abundance** | **Mean abundance** | **Mean visitation frequency** |
| --- | --- | --- | --- |
| **Ectatomminae** | | | |
| *Ectatomma tuberculatum* (Olivier, 1792) | 225 | 0.77±0.65 | 0.45±0.23 |
| **Formicinae** | | | |
| *Brachymyrmex* sp. | 360 | 5.13±2.48 | 0.20±0.10 |
| *Camponotus* *crassus* (Mayr, 1862) | 159 | 1.78±1.19 | 0.53±0.30 |
| *Camponotus rufipes* (Fabricius, 1775) | 42 | 1.11±0.99 | 0.47±0.41 |
| *Camponotus renggeri* (Emery, 1894) | 19 | 1.73 | 0.55 |
| *Camponotus ager* (Smith, 1858) | 3 | 0.25 | 0.25 |
| **Myrmicinae** | | | |
| *Crematogaster goeldii* (Forel, 1903) | 332 | 4.53±2.88 | 0.30±0.15 |
| *Cephalotes* sp. | 28 | 0.82±0.16 | 0.21±0.14 |
| **Ponerinae** | | | |
| *Neoponera* *villosa* (Fabricius, 1804) | 8 | 0.26±0.29 | 0.26±0.29 |
| **Pseudomyrmicinae** | | | |
| *Pseudomyrmex gracilis* (Fabricius, 1804) | 5 | 0.13±0.09 | 0.11±0.04 |
