# Supplementary material for: Pericarpial nectary-visiting ants do not provide fruit protection against pre-dispersal seed predators regardless of ant species composition and resource availability
Source: PLoS One. 2017 Dec 6;12(12):e0188445. doi: 10.1371/journal.pone.0188445 (PMC5718428; doi:10.1371/journal.pone.0188445)
Supplement: S2 Table — Abundance, relative frequency and mean number (±SD) of seed predators per fruit (n = 245 fruits) among Tocoyena formosa individual plants. (DOCX) [file pone.0188445.s002.docx]

| **Order** | **Abundance** | **Relative frequency (%)** | **Mean number/ fruit/plant** |
| --- | --- | --- | --- |
| **Lepidoptera** | 3 | 0.97 | 0.01±0.04 |
| **Diptera** | 23 | 7.5 | 0.11±0.24 |
| **Coleoptera** | 133 | 43.3 | 0.59±0.62 |
| **Hymenoptera** | 148 | 48.2 | 0.60±1.48 |
| **Total** | **307** | **100** | **1.30±1.62** |

**S2 Table. Pre-dispersal seed predators of Tocoyena formosa.** Abundance, relative frequency and mean number (±SD) of seed predators per fruit (n= 245 fruits).
