# Supplementary figures and images for: Pericarpial nectary-visiting ants do not provide fruit protection against pre-dispersal seed predators regardless of ant species composition and resource availability
Source: PLoS One. 2017 Dec 6;12(12):e0188445. doi: 10.1371/journal.pone.0188445 (PMC5718428; doi:10.1371/journal.pone.0188445)

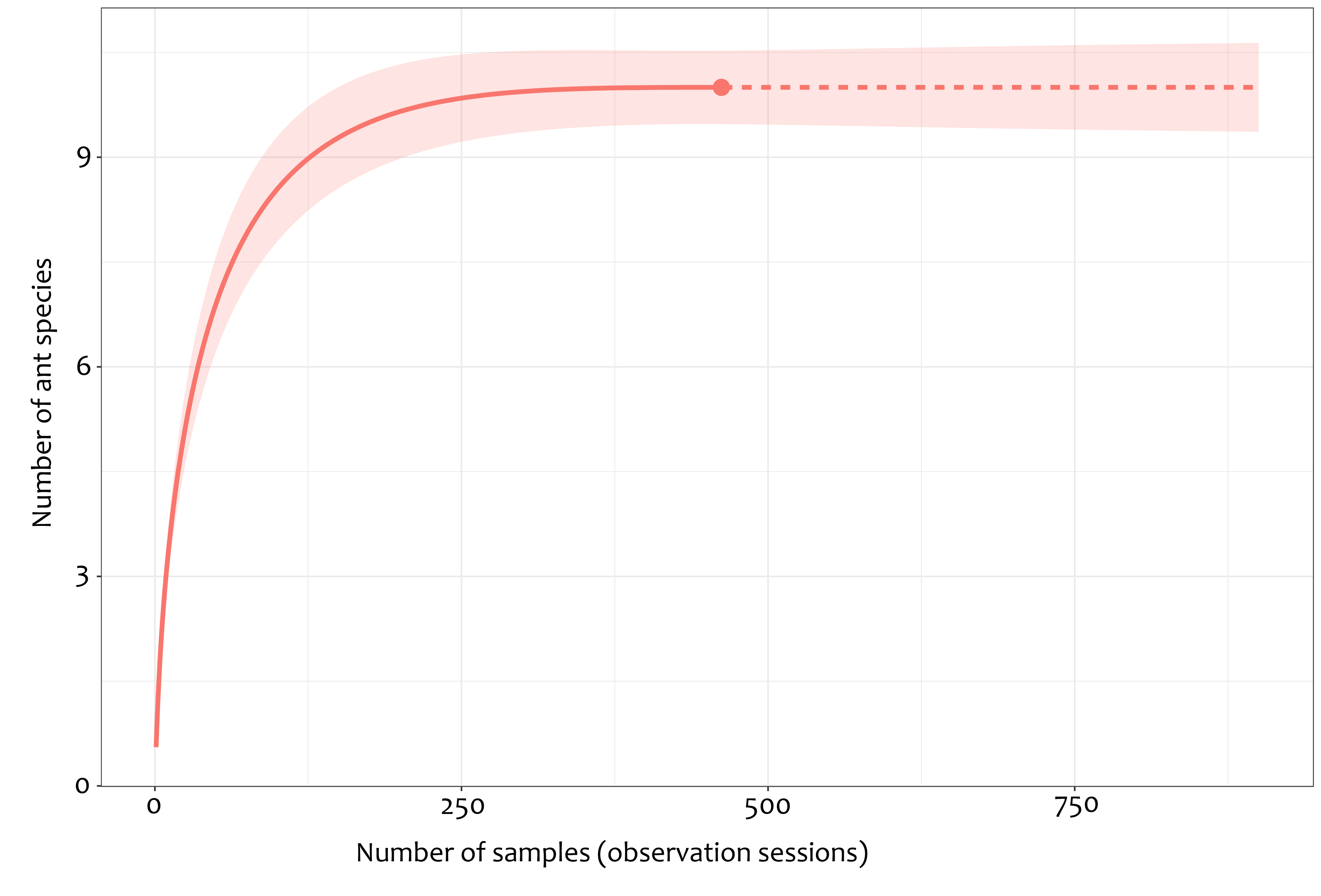

Supplement: S1 Fig — Solid and dotted lines represent rarefaction and extrapolation curves, respectively. Shaded area shows the 95% confidence intervals after 250 bootstraps. (TIF) [file pone.0188445.s003.tif]

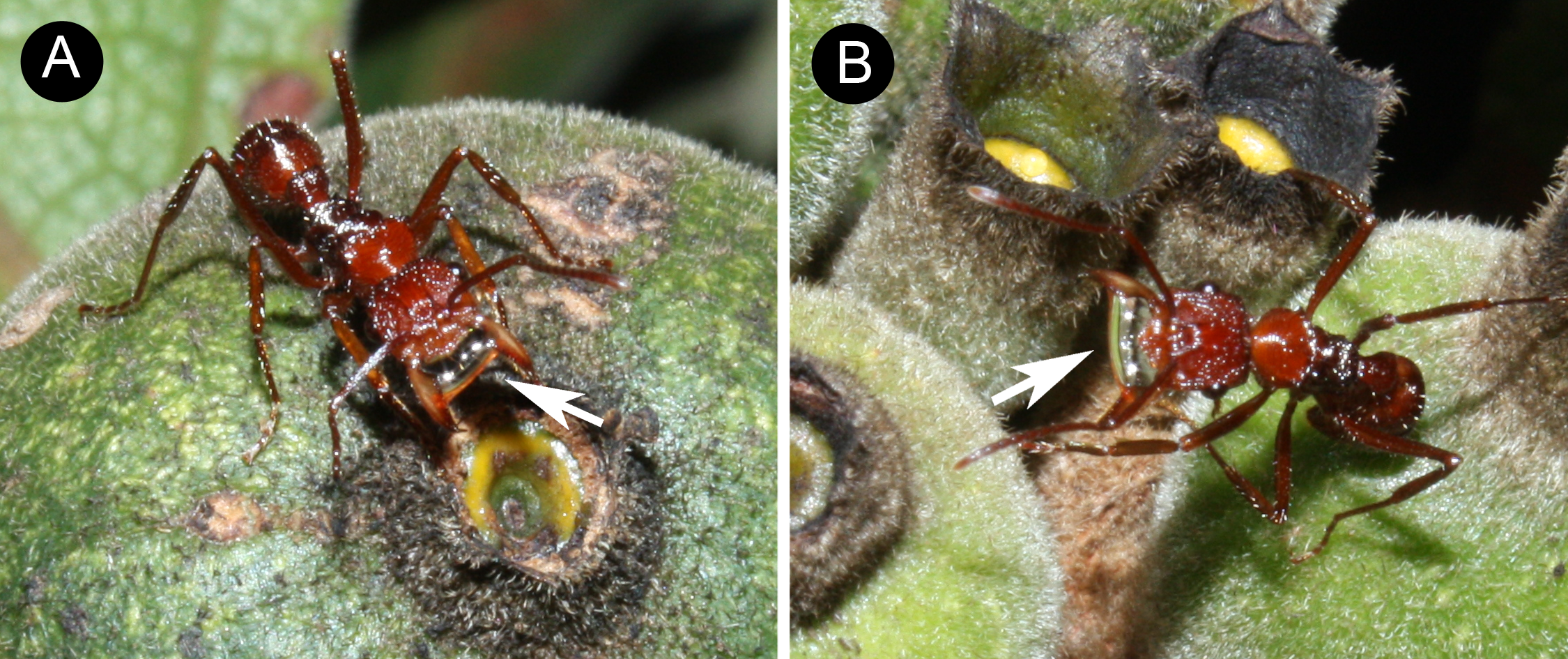

Supplement: S2 Fig — (A) and (B) show E. tuberculatum carrying a nectar droplet between its jaws (arrows). (TIF) [file pone.0188445.s004.tif]
